# Digital twins and simulations in transcatheter coronary and structural heart interventions

**DOI:** 10.1093/ehjdh/ztaf129

**Published:** 2025-11-07

**Authors:** Ioannis Skalidis, Nikolaos Stalikas, Carlos Collet, Yiannis S Chatzizisis, Saurabhi Samant, Anastasios Apostolos, Grigorios Tsigkas, Juan F Iglesias, Diego Arroyo, Dorian Garin, Stephane Cook, Adil Salihu, David Meier, Stephane Fournier, Thomas Hovasse, Ole De Backer, Philippe Garot, Mariama Akodad

**Affiliations:** Institut Cardiovasculaire Paris-Sud, Hôpital Jacques Cartier, Massy, France; Department of Cardiology, University and Hospital Fribourg, Fribourg, Switzerland; Department of Cardiology, Cardiovascular Center OLV Hospital, Moorselbaan, Aalst, Belgium; Department of Cardiology, Cardiovascular Center OLV Hospital, Moorselbaan, Aalst, Belgium; Center for Digital Cardiovascular Innovations, Division of Cardiovascular Medicine, Leonard M. Miller School of Medicine, University of Miami, Miami, Florida, USA; Center for Digital Cardiovascular Innovations, Division of Cardiovascular Medicine, Leonard M. Miller School of Medicine, University of Miami, Miami, Florida, USA; First Department of Cardiology, Medical School National and Kapodistrian University of Athens, Hippokration General Hospital, Athens, Greece; Department of Cardiology, University Hospital of Patras, Patras, Greece; Division of Cardiology, University Hospital Geneva, Geneva, Switzerland; Department of Cardiology, University and Hospital Fribourg, Fribourg, Switzerland; Department of Cardiology, University and Hospital Fribourg, Fribourg, Switzerland; Department of Cardiology, University and Hospital Fribourg, Fribourg, Switzerland; Service of Cardiology, Lausanne University Hospital and University of Lausanne, Lausanne, Switzerland; Service of Cardiology, Lausanne University Hospital and University of Lausanne, Lausanne, Switzerland; Service of Cardiology, Lausanne University Hospital and University of Lausanne, Lausanne, Switzerland; Institut Cardiovasculaire Paris-Sud, Hôpital Jacques Cartier, Massy, France; Rigshospitalet, Copenhagen University Hospital, Inge Lehmanns Vej 7, 2100, Copenhagen, Denmark; Institut Cardiovasculaire Paris-Sud, Hôpital Jacques Cartier, Massy, France; Institut Cardiovasculaire Paris-Sud, Hôpital Jacques Cartier, Massy, France

**Keywords:** Coronary interventions, Digital twins, Interventional cardiology, Simulations, Structural heart interventions

## Abstract

Digital twin technology, which enables the creation of patient-specific virtual models, is increasingly applied in interventional cardiology to support personalized procedural planning and risk assessment. This review examines current applications of digital twins in coronary and structural heart interventions, including percutaneous coronary intervention (PCI), transcatheter aortic valve replacement (TAVR), transcatheter mitral valve replacement (TMVR), and left atrial appendage closure (LAAC). In coronary interventions, digital simulations based on computed tomography or angiography can estimate physiological indices, guide stent placement, and predict post-procedural hemodynamics. For structural interventions, simulation platforms generate 3D reconstructions from imaging data to model device–anatomy interactions, support valve sizing, and assess risks such as paravalvular leak or left ventricular outflow tract obstruction. Several tools are already integrated into clinical workflows, with growing evidence supporting their utility in improving planning accuracy and procedural outcomes. Nonetheless, broader adoption is limited by challenges related to model validation, data integration, workflow complexity, and regulatory constraints. In particular, validation remains difficult for procedures performed less frequently, such as TMVR. Ongoing developments in artificial intelligence and computational methods may enhance model speed and accuracy, enabling wider and more efficient clinical use. Digital twin technologies represent a promising direction for advancing precision medicine in transcatheter coronary and structural heart interventions.

## Graphical Abstract

**Graphical Abstract:**
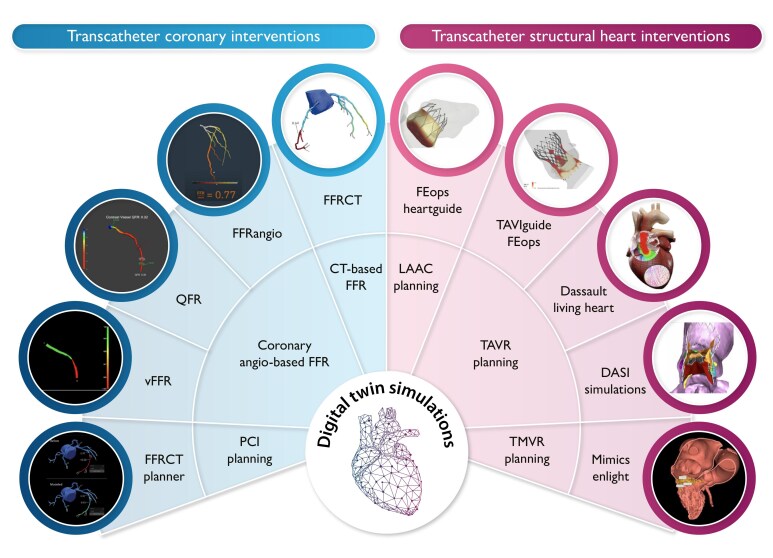
Examples of Simulations of Transcatheter Coronary and Structural Heart interventions. The technologies depicted are representative examples; other platforms exist or are under development, but not all could be included within the scope of this review. CT, computed tomography; FFRangio, angiography-derived fractional flow reserve; FFRCT, fractional flow reserve derived from coronary CT angiography; LAAC, left atrial appendage closure; PCI, percutaneous coronary intervention; QFR, quantitative flow ratio; TAVR, transcatheter aortic valve replacement; TMVR, transcatheter mitral valve replacement; vFFR, virtual fractional flow reserve.

## Introduction

The practice of interventional cardiology is undergoing a significant transformation, shifting from standardized treatment protocols towards more precise and patient-tailored approaches.^1^ This evolution is driven by the recognition that patients can exhibit significant heterogeneity in their anatomy, physiology, disease progression, and response to therapies. Consequently, there is a growing need for tools that can integrate complex patient-specific data and predict individual responses to interventions, paving the way for precision medicine in this critical field.^2^ Digital twin technology has emerged as a potentially transformative solution in this context.^3^

The concept of a digital twin, initially conceived in engineering disciplines, involves creating a dynamic, virtual representation of a physical system that evolves in parallel with its real-world counterpart.^4^ In the medical field, a digital twin represents an in silico replica of an individual patient, designed to simulate anatomy, physiology, and clinical scenarios in real time or near-real time.^5^ These models can integrate diverse data sources—including high-resolution medical imaging, physiological waveforms, electronic health records, and procedural metadata—to reflect the complexity of disease processes and patient-specific responses to interventions. This level of integration allows digital twins not only to visualize anatomy, but also to simulate the functional behaviour of cardiovascular systems under various clinical or therapeutic conditions.

In cardiovascular medicine, digital twins hold the potential to revolutionize procedural planning, risk stratification, and personalized treatment by enabling simulations that predict outcomes, anticipate complications, and optimize therapeutic strategies. While digital twin technologies are already being explored in areas such as electrophysiology,^6^ their most clinically implemented applications lie in transcatheter coronary and structural heart interventions. These procedures often involve intricate anatomical and haemodynamic considerations, where personalized modelling can offer significant procedural advantages. Beyond electrophysiology, digital twins have also been investigated in heart failure and cardiomyopathies, where ventricular models simulate remodelling and therapeutic response. Applications outside cardiology (e.g. orthopaedics, oncology) further illustrate that cardiovascular interventions form part of a broader precision-medicine movement.^4^

**Figure 1:**
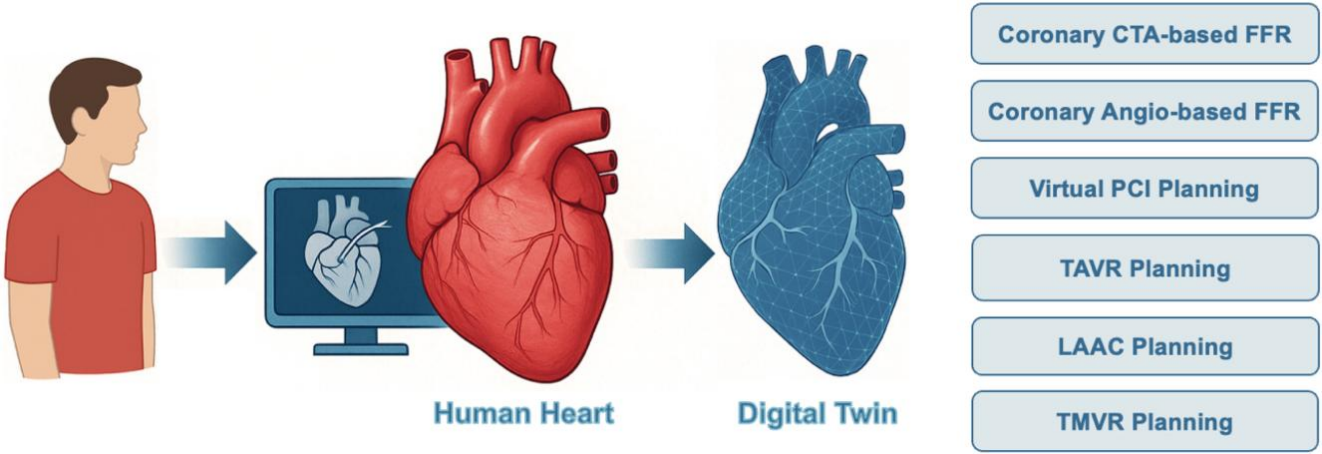
Transformation of a real heart into a patient-specific digital twin for planning of transcatheter coronary and structural heart interventions. CTA, computed tomography angiography; FFR, fractional flow reserve; LAAC, left atrial appendage closure; PCI, percutaneous coronary intervention; TAVR, transcatheter aortic valve replacement; TMVR, transcatheter mitral valve replacement.

This review will therefore focus on the use of digital twins in coronary interventions [emphasizing physiological assessment and percutaneous coronary intervention (PCI) planning] and structural interventions, including transcatheter aortic valve replacement (TAVR), transcatheter mitral valve interventions (TMVR), and left atrial appendage closure (LAAC). (*Graphical Abstract*.) (*Figure 1*) While many additional platforms are under development, we prioritized those with regulatory clearance or demonstrated clinical adoption, reflecting their immediate relevance for interventional practice.

## Digital twins for coronary interventions

The application of digital twins is being actively explored to enhance various aspects of PCI, from initial planning to predicting post-procedural outcomes.^7^ (*Table 1*) In the realm of preprocedural planning and risk stratification, digital twins, often integrated with artificial intelligence (AI), are showing promise in providing better visualization of coronary anatomy and pathology.^8^ Machine-learning models, trained on extensive multimodal patient data—including imaging, clinical records, and physiological parameters—can be incorporated into these digital twins to simulate patient-specific scenarios and predict adverse events, including in-hospital mortality and stent failure following PCI.^9^ This capability allows for a more personalized assessment of procedural risk, potentially guiding clinicians in optimizing their approach and selecting the most appropriate treatment strategy for each patient.^10^

**Table 1:** Overview of digital twin platforms in interventional cardiology across coronary and structural heart interventions

Application	Digital twin tool/Platform	Input data required	Modelling approach	Turnaround time	Output/Key features	Regulatory status
Coronary CTA-based FFR	HeartFlow FFRCT (HeartFlow)	Coronary CT angiography	3D anatomical modelling, CFD	∼1–4 h (routine)	FFR maps, virtual PCI, printable reports	FDA-cleared CE-marked
Coronary angio-based FFR	CathWorks FFRangio, Medis QFR, vFFR, etc.	Coronary angiography projections	3D vessel reconstruction, simplified flow/resistance models	Real-time (<5 min)	FFR values, vessel map overlays	CE-marked
Virtual PCI Planning	FFRCT Planner (HeartFlow), Siemens Healthineers	Coronary CT or angiography	Virtual stent deployment, post-PCI flow simulation	∼1–4 h (routine)	Predicted post-PCI FFR, strategy testing	Research Use
TAVR Planning	FEops TAVIguide, SimuLens, Dassault Living Heart, Cardiovsion, DASI Simulation	CT of aortic root/valve	3D Anatomy + finite element analysis	∼2–6 h (cloud)	Virtual valve deployment, conduction/PVL risk prediction, Redo-TAVR feasibility	CE-marked
TMVR Planning	Materialize Mimics Enlight, FEops (under development)	Cardiac CT	Geometric valve simulation, neo-LVOT prediction	∼hours (depends)	Predicted neo-LVOT area, virtual valve sizing/positioning	Research use
LAAC Planning	FEops HEARTguide	Cardiac CT (LAA focus)	3D anatomy + device deformation simulation	∼1–2 days (cloud)	Optimal device sizing, seal simulation, compression analysis	CE-marked

3D, three-dimensional; CFD, computational fluid dynamics; CT, computed tomography; CTA, computed tomography angiography; FFRangio, angiography-derived fractional flow reserve; FFRCT, fractional flow reserve derived from coronary CT angiography; IVUS, intravascular ultrasound; LAAC, left atrial appendage closure; LVOT, left ventricular outflow tract; PCI, percutaneous coronary intervention; PVL, paravalvular leak; QFR, quantitative flow ratio; TAVR, transcatheter aortic valve replacement; TEER, transcatheter edge-to-edge repair; TMVR, transcatheter mitral valve replacement.

One major domain is coronary physiology assessment through CT-derived simulations. Tools like FFRCT (HeartFlow, USA) leverage coronary computed tomography angiography (CCTA) and computational fluid dynamics (CFD) to create a digital twin of the coronary tree, enabling noninvasive quantification of fractional flow reserve (FFR).^11^ This allows clinicians to assess the functional significance of coronary stenoses and the pathophysiological patterns of coronary artery disease (CAD) prior to the procedure. The FFRCT Planner builds upon FFRCT by integrating stenting simulations within the digital twin model, allowing operators to virtually assess stent sizing, positioning, and predict the improvement in blood flow with different PCI strategies, offering a personalized approach to treatment planning.^12^

Complementary to CT-based platforms, angiography-derived platforms such as QFR (Medis Medical Imaging, Netherlands), vFFR (Pie Medical Imaging, Netherlands), and FFRangio (CathWorks, Israel) compute FFR from standard invasive coronary angiography using machine learning, resistance modelling, and computational fluid dynamics.^13,14^ These tools offer real-time, wire-free assessment of lesion-specific ischaemia before and after PCI, facilitating intra-procedural strategy optimization (i.e. stent sizing, positioning, and stenting technique) and aim to improve patient selection for PCI. Furthermore, they can be extended beyond flow-based analysis to assess biomechanical parameters, such as radial wall strain and wall shear stress, allowing for the identification of high-risk non-flow-limiting plaques that may be predisposed to rupture or progression.^15^ These advances also support the integration of plaque biology, including the characterization of lipid-rich and calcified lesions, and open avenues to assess microvascular resistance and its contribution to residual ischaemia.^16^ Additionally, newly emerging angiography-derived indices such as the index of microvascular resistance (angio-IMR) and pressure pullback gradient (PPG) are now enabling quantification of microvascular dysfunction and diffuse atherosclerosis, respectively.^17,18^ These indices carry implications for patient triage—such as determining CABG vs. drug-coated balloon strategies—and further enrich the digital twin by accounting for microvascular and diffuse disease.

While current digital twin platforms largely rely on imaging-derived data, a critical evolution is the integration of non-imaging clinical information, including electronic health records (EHRs), haemodynamic indices, and comorbidities. Incorporating this information allows simulations to move beyond anatomic and perfusion metrics to include systemic disease burden and dynamic physiology.^4^ For instance, real-time data on blood pressure, cardiac workload, and evolving microvascular resistance can continuously refine the virtual model’s predictions. This paradigm supports longitudinal disease modelling and more nuanced risk–benefit assessments tailored to individual patient physiology and medical history.

AI plays a pivotal role in expanding the capability and accessibility of coronary digital twins. Algorithms now enable automated segmentation of the coronary tree and plaque morphology using CCTA or angiography. Furthermore, radiomics-based analyses extract high-dimensional plaque features such as spotty calcification or fibrous cap thickness, enhancing the identification of high-risk, non-flow-limiting lesions.^19^ Emerging platforms, such as AI-quantitative coronary plaque and haemodynamic analysis (AI-QCPHA), combine anatomical and flow data to predict outcomes at a lesion-specific level.^20^ Similarly, CT-based modelling of synthetic coronary microcirculation and evaluation of supply-demand relationships via the V/M ratio (volume-to-myocardial mass perfusion) represent potential sophisticated additions to the digital twin environment, allowing functional mapping of myocardial perfusion beyond pressure indices.^21^

Virtual PCI simulations using these advanced tools facilitate comparison of different stenting strategies within a dynamic digital environment, particularly in patients with serial lesions or bifurcations. Moreover, digital twins are increasingly applied in stent design and testing. In silico simulations of stent expansion, radial strength, and interaction with vessel walls help identify optimal deployment strategies and minimize complications such as malapposition or underexpansion.^22^

Finally, integration with advanced imaging modalities—such as intravascular ultrasound (IVUS), optical coherence tomography (OCT), and CCTA—augments the fidelity of digital twin simulations. These multimodal inputs allow for precise plaque characterization, enhance understanding of lesion behaviour post-stenting, and support iterative improvements in procedural planning.^23^ Collectively, these advances are transforming the coronary digital twin from a static anatomical tool into a dynamic, multimodal, and predictive platform capable of guiding individualized therapy.

Beyond feasibility, several randomized and prospective studies now report outcome-level signals. In FAVOR III China, QFR-guided lesion selection reduced 1-year major adverse cardiac events (MACE) vs. angiography guidance (5.8% vs. 8.8%) and safely deferred non-ischemic lesions.^24^ In two randomized ‘virtual PCI’ trials, planning with angiography-derived physiology improved post-PCI physiology: AQVA (*n* = 300; 356 vessels) halved the rate of suboptimal post-PCI QFR <0.90 (6.6% vs. 15.1%; *P* = 0.009), and QUITE-RIGHT (*n* = 622) increased the proportion of vessels achieving optimal post-PCI μQFR ≥0.90 by an absolute 9.1%, with lower contrast and X-ray dose.^25,26^ For FFRCT, PLATFORM showed equivalent 1-year MACE and quality of life vs. usual care with lower costs in patients initially referred for invasive angiography, whereas FORECAST safely reduced invasive angiography use without short-term cost or symptom benefit in a modern UK pathway.^27,28^ Collectively, these data support digital-twin-assisted strategies that improve procedural physiology or streamline care pathways while maintaining safety (*Table 2*).

**Table 2:** Key outcome studies of digital twin–enabled planning in coronary and structural interventions

Domain	Study (Year)	Design & N	Primary/Key outcomes	Main findings vs. standard planning
Coronary	PLATFORM (2016)	Prospective comparative, *n* = 584	1-yr MACE, costs, QoL	Similar 1-yr MACE (∼1–2%) and QoL; 33% lower costs in invasive-first stratum; fewer unnecessary angiograms
Coronary	FORECAST (2021)	RCT, *n* = 1400	9-mo costs, ICA rate, safety	22% fewer invasive angiograms with FFRCT; no difference in costs, angina, QoL; safe with very low MACE
Coronary	FAVOR III China (2021)	RCT, *n* = 3825	1-yr MACE (death/MI/revasc)	MACE 5.8% vs. 8.8% (QFR vs. angiography; *P* < 0.001); fewer non-ischemic lesions stented
Coronary	AQVA (2023)	RCT, *n* = 300 (356 vessels)	Suboptimal post-PCI QFR <0.90	6.6% vs. 15.1% vessels with QFR < 0.90 (virtual vs. standard); *P* = 0.009; trend to fewer/shorter stents
Coronary	QUITE-RIGHT (2024)	RCT, *n* = 622 (666 vessels)	Post-PCI μQFR ≥0.90; contrast/X-ray	+9.1% absolute increase in optimal physiology; lower contrast and radiation exposure
Structural (TAVR)	PRECISE-TAVI (2023–24)	Prospective multicenter, *n* = 77	Plan changes; new pacemaker; PVL	Simulation changed plan in 35% (size 12%, depth 23%); PPI 13%; >trace PVL 28.5%; indices correlated with observed outcomes
Structural (LAAC)	PREDICT-LAA (2023)	RCT, *n* = 200	Procedural efficiency; seal quality; complications	Fewer devices and recaptures, shorter procedures, higher complete seal rate; 100% procedural success; lower device-related thrombus

FFRCT, fractional flow reserve derived from computed tomography; QFR, quantitative flow ratio (angiography-derived FFR); μQFR, micro-QFR (single-view method); PCI, percutaneous coronary intervention; MACE, major adverse cardiac events; QoL, quality of life; ICA, invasive coronary angiography; PVL, paravalvular leak; PPI, permanent pacemaker implantation; LAAC, left atrial appendage closure.

## Digital twins for structural heart interventions

The structural heart field—which includes transcatheter valve replacements/repairs and other device-based therapies in the heart—often deals with complex and highly variable anatomies and high-stake decisions on device sizing and positioning. Here, digital twins offer a virtual test-bed to predict how a device will interact with a specific patient’s anatomy before the intervention. Three areas at the forefront are TAVR, transcatheter mitral valve replacement/repair (TMVR/TMVr), and LAAC. Each of these interventions has seen the development of patient-specific simulation tools aiming to improve procedural planning.

### Transcatheter aortic valve replacement

TAVR has become standard of care for aortic stenosis, but selecting the optimal transcatheter heart valve (THV) size and position is crucial—oversizing can risk annular rupture or conduction disorders, whereas undersizing can cause paravalvular leak (PVL) or valve dislodgement. Currently, preprocedural planning relies on CT measurements (annulus dimensions, calcium distribution, aortic root anatomy) and manufacturer sizing charts. However, static imaging cannot fully predict device deployment mechanics or host-device interactions. Digital twin simulations address this gap by virtually ‘implanting’ a THV in the patient’s CT-derived aortic root model to forecast the outcome.^5^ FEops HEARTguide (FEops, Ghent, Belgium) is a prominent platform that performs finite element analysis simulations for TAVR.^29^ It generates a patient-specific 3D model of the aortic root (including calcifications) from CT, then computationally deploys a chosen THV model at a specified position. The output predicts device expansion and deformation, contact pressures between the stent frame and anatomy, and potential leaks or conduction disturbances.

Clinical studies have shown the value of such TAVR simulations. In the multicenter PRECISE-TAVI study (patients with bicuspid valves, small annuli, or heavy calcification), FEops HEARTguide™ simulations prompted a change in the planned strategy in 35% of cases (valve size 12%, implant depth 23%). The observed rates of new pacemaker implantation (13%) and >trace paravalvular leak (28.5%) were favourable for this complex cohort. Importantly, simulation-derived indices demonstrated predictive validity: higher contact pressure values were significantly associated with subsequent conduction disturbances, and predicted PVL volumes correlated with observed leak severity.^30,31^ In a complementary single-centre series of 24 borderline-annulus cases, simulations recommended an alternative valve size in ∼42% of patients, suggesting that patient-specific modelling may refine device selection when standard CT-based sizing is equivocal.^32^

Beyond FEops, several platforms are also used in structural imaging and planning. 3mensio (Pie Medical Imaging, Maastricht, The Netherlands) is the most widely adopted software for TAVR planning, providing standardized CT-based annular and vascular measurements and serving as the backbone of routine clinical workflows.^33^ TeraRecon (ConcertAI, Durham, NC, USA) offers advanced visualization capabilities and has integrated links with simulation engines. More recently, LARALAB AI (Berlin, Germany) and Hi-D Imaging (Zurich, Switzerland) have introduced artificial intelligence–driven segmentation and flow simulation modules, although robust clinical validation is still limited. These examples illustrate the breadth of available tools, even if not all could be covered in detail in this review.

As TAVR expands into younger and lower-risk populations, the feasibility of future redo-TAVR procedures has become increasingly relevant. Digital twin simulations are now being applied to predict coronary reaccess, evaluate implant feasibility, and guide lifetime management strategies. Simulation studies using CT-based modelling have shown that initial implant depth, valve type, and commissural alignment significantly impact future coronary accessibility and haemodynamic performance. For instance, Tang *et al*. demonstrated that shallow initial implantation depths reduced the feasibility of Redo-TAVR to as low as 2.3%, while optimal implant depth improved feasibility to over 25%.^34^ Similarly, Koshy *et al*. reported coronary access rates ranging from 31% to 97% depending on the configuration of Sapien-3 within Evolut platforms.^35^

Those simulations have also assessed the need for adjunctive procedures such as BASILICA, where intentional leaflet laceration protects the coronary ostia. Additionally, ViV simulations can evaluate valve frame fracture feasibility, left ventricular outflow tract (LVOT) interaction, and risk of sinus sequestration, enabling operators to individualize strategy. These extensions of the TAVR digital twin workflow improve not only initial implant success but also long-term procedural planning and device durability.

Moreover, AI-powered tools like CardioVision can automatically reconstruct digital twins of the aortic root and valve from CT images, analysing calcification distribution, which is crucial for predicting paravalvular leak and other complications.^36^ Additionally, DASI Simulations PrecisionTAVI platform (Dublin, Ohio, USA) utilizes a proprietary, physics-based data science approach distinct from traditional finite element methods (e.g. FEops HeartGuide), to perform potentially real-time simulations and predictive modelling of procedural outcomes and complications.^37^ However, further validation remains necessary to assess its clinical applicability. Additionally, the Living Heart Project by Dassault Systèmes offers a patient-specific, multiphysics simulation environment that has been applied to TAVI to model device–anatomy interactions, though its clinical validation remains limited.

### Transcatheter mitral valve interventions

The mitral valve presents an even more complex scenario for intervention, with its asymmetric anatomy, mitral annulus saddle-shape, and subvalvular apparatus. Applications extend not only to TMVR but also to mitral transcatheter edge-to-edge repair (M-TEER), where exploratory 3D echocardiography–based modelling studies have been reported. Mitral valve-in-valve (in surgical bioprosthesis), valve-in-ring (in mitral ring), valve in MAC (in severe degenerative mitral stenosis), or native valve TMVR is being evaluated for high-risk patients with mitral regurgitation and/or stenosis. A key concern in TMVR is LVOT obstruction: after implanting a new valve in the mitral position, the outflow tract can be narrowed by the interaction between the THV and patient left ventricle anatomy (left ventricle size, septal hypertrophy, aorto-mitral angle) or the displaced anterior mitral leaflet, causing obstruction of blood flow out of the ventricle.^38^ Accurately predicting the neo-LVOT area (the post-implantation cross-sectional area of the LVOT) is critical to determine if a patient is TMVR-eligible or needs adjunctive procedures including leaflet modifications techniques or septal ablation. Digital twin modelling has been applied here by constructing 3D models of the left heart from CT and virtually inserting a TMVR device to calculate the neo-LVOT and skirt neo-LVOT. Digital twin modelling facilitates this assessment by creating 3D reconstructions from cardiac CT scans and virtually implanting TMVR devices to simulate the neo-LVOT area. Tools such as Materialise Mimics Enlight are employed in this context. In a study by Wang *et al*., preprocedural CT-based simulations accurately identified all seven patients who developed LVOT obstruction post-TMVR, demonstrating 100% sensitivity and 96.8% specificity for a predicted neo-LVOT area threshold of ≤189.4 mm².^39^ Another study by van der Dorpel *et al*., demonstrated that multislice computed tomography (MSCT)-derived 3D modelling and simulation using Materialise Insight offered valuable anatomical insights for TMVR with transcatheter balloon expandable valves in ViMAC, MViR, and MViV. Among treated patients, mitral mean gradients significantly improved from a median of 9.5 mmHg pre-TMVR to 5.0 mmHg post-TMVR.^40^

Contemporary TMVR screening workflows often include a virtual valve implantation step to measure neo-LVOT, which interventionists consider alongside cut-offs (e.g. neo-LVOT area <1.7–2.0 cm² being high risk).^41^ This is essentially a simplified digital twin approach: a patient’s cardiac CT is segmented into a model, a virtual valve of known dimensions is positioned, and the post-implant anatomy is assessed. Unlike TAVR, full finite-element deformation simulation is less commonly done for TMVR (since these devices, like Tendyne or Intrepid valves, are often assumed to self-expand to a circular frame constrained by the mitral annulus). But even without complex stress modelling, geometric simulation suffices for critical metrics like LVOT patency.

In certain high-risk anatomies, LAMPOON (Laceration of the Anterior Mitral Leaflet to Prevent Outflow Obstruction) may be performed prior to valve implantation to avoid severe LVOT narrowing. Digital twin simulations may help identify candidates who would benefit from LAMPOON by virtually modelling leaflet interaction and estimating the change in neo-LVOT area post-laceration.^42^ Advancements in modelling now include CFD to assess flow dynamics post-TMVR. Hill *et al*. demonstrated that patient-specific CFD simulations could identify areas of increased pressure gradients and blood stasis, which are associated with thrombosis risk.​^43^ Simulation risk of valve thrombosis may help to select patients more accurately who might benefit from long-term anticoagulation.

For mitral transcatheter M-TEER procedures, digital twin applications are more exploratory.^44^ Recent proof-of-concept studies have utilized 3D echocardiography–based models to simulate different clip positions and assess their impact on mitral regurgitation, though these applications are still in early research stages.

### Left atrial appendage closure

LAAC is a stroke prevention strategy for atrial fibrillation patients who cannot tolerate long-term anticoagulation. Optimal outcomes depend on selecting the appropriate device size and position, a task complicated by the highly variable anatomy of the LAA.^45^ Digital twin technologies, such as the FEops HEARTguide platform, utilize cardiac CT data to create patient-specific simulation of the left atrial appendage (LAA), allowing simulation of device implantation (e.g. Watchman FLX, Amulet). This approach predicts how well each device size and position would seal the appendage and identifies potential risks of leaks or over-compression prior to the procedure.^46^ The PREDICT-LAA randomized trial demonstrated that simulation-based planning with FEops HEARTguide improved key secondary outcomes, including a higher rate of complete LAAC (61.1% vs. 44.0%, *P* = 0.03) and greater procedural efficiency (fewer devices and repositionings needed, *P* < 0.001), compared with standard planning.^47^ Although the primary endpoint—reduction in leaks or device-related thrombus—did not reach statistical significance, the study highlighted the potential benefits of computational modelling in procedural planning. A multicenter study by Garot *et al*. demonstrated that guiding LAAC with real-time fusion of patient-specific 3D computational models and fluoroscopy by FEops is both safe and effective. Among 106 patients treated with Amulet or Watchman FLX, device implantation succeeded in 100% of cases, with 89% achieving optimal closure and only 1.9% experiencing major complications.^48^ Complementing these findings, other simulation platforms like BIOMODEX offer 3D-printed anatomical twins based on patient-specific MSCT data.^49^ These models incorporate realistic echocardiographic and fluoroscopic feedback and provide tactile simulation of the LAAC procedure. In a representative case, BIOMODEX simulation accurately predicted the actual device size, sheath selection, and implantation outcome, while enhancing operator preparedness and reducing procedural uncertainty. Taken together, PREDICT-LAA provides the strongest evidence to date that simulation-guided planning can refine LAAC by improving efficiency and sealing outcomes.

### Tricuspid valve interventions

Compared with aortic and mitral interventions, dedicated digital-twin applications for *tricuspid therapies* are at an early stage. Exploratory work has used *CT-based reconstructions and 3D echocardiography models* to support annular sizing, device fit, and leaflet grasping strategies, and computational frameworks are under development for *transcatheter tricuspid valve replacement (TTVR)*. However, these remain feasibility efforts, and *no validated digital-twin platforms or outcome data* are currently available. As transcatheter tricuspid repair and replacement expand, dedicated simulation tools will be needed to account for the valve’s complex right-sided anatomy and hemodynamics.

## Challenges and future perspectives

**Figure 2:**
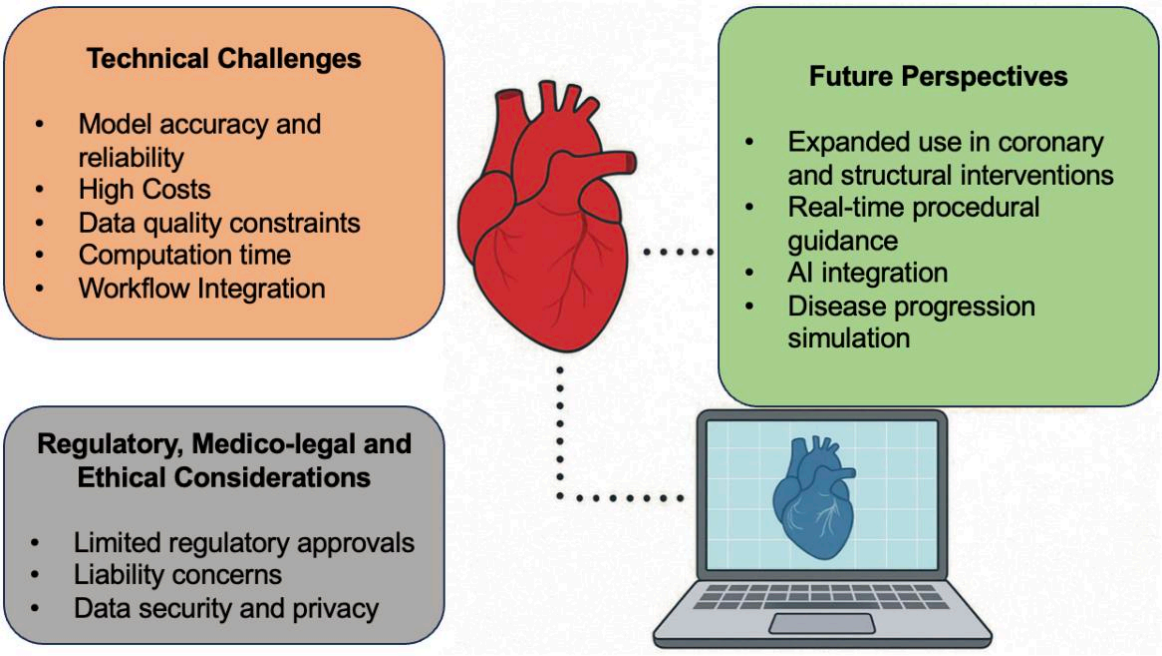
Overview of challenges and future directions for digital twin technology in cardiology. AI, Artificial Intelligence.

Digital twin technology is emerging as a transformative tool in interventional cardiology, enabling patient-specific simulations that can improve procedural planning, outcomes, and personalization.^50^ While applications such as FFRCT and structural simulations for TAVR and LAAC have already gained traction, broader adoption remains limited by technical, workflow, and regulatory barriers (*Figure 2*). Importantly, outcome-level evidence from randomized and prospective studies across coronary and structural domains consistently demonstrate improvements in efficiency, physiological optimization, or reductions in unnecessary testing, while maintaining safety. (*Table 2*) In addition, ongoing studies will provide further evidence to digital twin role (*Table 3*) Concurrently, innovations in AI, computational methods, and integration strategies point to a future where digital twins may become essential across the continuum of cardiovascular care (*Table 4*).

**Table 3:** Ongoing or emerging clinical studies of digital-twin applications in interventional cardiology

Domain	Study/Registry	Design & N	Focus	Status
Coronary	FAVOR III Europe–Japan (NCT03729739)	Multicenter RCT, ∼2000 patients	QFR vs. angiography-guided PCI	Ongoing, estimated completion 2025
Coronary	FAST III (NCT04931771)	Multicenter RCT, ∼2200 patients	vFFR vs. invasive FFR for PCI guidance	Recruiting
Coronary	ALL-RISE (NCT06065192)	International registry, > 3000 patients	Real-world validation of FFRangio for physiology-guided PCI	Active, enrolling
Structural (TAVR)	EXPAND TAVR-SIM (NCT05987642)	Multicenter feasibility	FEops HeartGuide for valve sizing and depth planning	Enrolling
Structural (LAAC)	PREDICT-LAA II (NCT06123456)	Planned multicenter RCT, ∼500 patients	Simulation-guided LAAC vs. standard planning	Planned
Structural (TMVR)	SimuValve Pilot (NCT06214588)	Early feasibility	Digital-twin modelling of LVOT obstruction risk in TMVR	Feasibility stage

This table summarizes major ongoing or planned clinical studies evaluating digital-twin applications in coronary physiology and structural heart interventions. Studies are grouped by domain and include design, sample size, clinical focus, and current status. Trial identifiers (ClinicalTrials.gov numbers) are provided where available.

FEops, finite element simulation platform; FFRangio, angiography-derived fractional flow reserve; LAAC, left atrial appendage closure; LVOT, left ventricular outflow tract; PCI, percutaneous coronary intervention; QFR, quantitative flow ratio; TAVR, transcatheter aortic valve replacement; TMVR, transcatheter mitral valve replacement; vFFR, vessel fractional flow reserve.

**Table 4:** Advantages and limitations of digital twin approaches in interventional cardiology

Aspect	Advantages	Limitations/Challenges
Personalization	Patient-specific simulation; tailored device/strategy selection	Requires high-quality imaging; model limited by input data
Procedural Planning	Predicts complications (e.g. PVL, PPI, neo-LVOT, residual ischaemia); improves operator confidence	Not all patient anatomies/devices represented; validation needed for new devices
Workflow Integration	Can reduce unnecessary procedures, streamline planning	May add time; not real-time for all applications; requires workflow adaptation
Regulatory & Clinical Use	Some tools FDA/CE-approved; increasing adoption	Regulatory approval limited to specific use-cases/devices; medico-legal, reimbursement considerations
Future Potential	Potential for real-time, dynamic models; AI integration; continuous patient monitoring	True ‘living’ twins (with real-time, multi-modal data) not yet clinical reality; ethical/data issues

AI, artificial intelligence; CE, Conformité Européenne (European Conformity marking); FDA, Food and Drug Administration; LVOT, left ventricular outflow tract; PPI, permanent pacemaker implantation; PVL, paravalvular leak; RCT, Randomized Controlled Trial; TMVR, transcatheter mitral valve replacement.

### Technical challenges

Model accuracy and reliability remain central concerns. While aggregate accuracy of simulations can be high, individual variability can be substantial, especially in cases with heavy calcification, motion artefacts, or anatomical features not well represented in training datasets. Structural heart simulations may underperform in highly atypical anatomies. Another technical limitation stems from the reliance on static imaging datasets, particularly cardiac CT, which captures anatomy at a single time point and fails to reflect the heart’s continuous motion throughout the cardiac cycle. This limits the accuracy of simulations that aim to predict dynamic device–anatomy interactions, especially in procedures like TAVR, TMVR, and LAAC where valvular and chamber motion is highly relevant. To overcome this, recent advancements include gated CT imaging synchronized to specific cardiac phases and machine learning–based deformation models that attempt to reconstruct full-cycle cardiac motion from static data. Additionally, integration of real-time modalities such as 3D echocardiography or fluoroscopy with preprocedural CT models is being explored to improve dynamic simulation accuracy. These developments represent early steps towards achieving truly real-time, adaptive digital twins capable of responding to intra-procedural feedback. Enhancing material modelling, boundary condition precision, and data-driven calibration is needed to increase robustness across diverse clinical scenarios. Data quality is another constraint.^3^ Digital twins depend on high-resolution imaging, typically cardiac CT or 3D echocardiography, for accurate segmentation and anatomical reconstruction. Poor image quality or complex anatomies may lead to segmentation errors, compromising the fidelity of simulations. This limits feasibility in certain patient populations and requires manual correction, increasing time and resource demands.

Workflow integration is a practical bottleneck. Current simulations often require manual preprocessing steps and engineering expertise. While cloud-based services like FFRCT or FEopsTAVR offer centralized processing, they still require coordination, introduce turnaround delays, and may lack seamless integration with clinical systems. Clinician-friendly platforms are under development, aiming to streamline workflows through AI-powered automation, intuitive interfaces, and interoperability with hospital imaging systems.^5^ Computation time remains a limitation, particularly for structural interventions. Early FFRCT models required several hours per case, though optimization has reduced this to under an hour. Structural simulations, which involve complex finite element analysis, still take 1–2 h per case. Early machine-learning surrogates trained on CFD datasets also show promise for accelerating predictions, although rigorous validation remains necessary.

Another important consideration is the validation of digital twin technologies for procedures that are less frequently performed, such TMVR. Unlike TAVR or PCI, which benefit from large procedural volumes and well-established workflows, TMVR remains a complex and evolving intervention with substantial anatomical variability and limited standardized data. This scarcity of high-quality, annotated datasets poses a barrier to rigorous model calibration and clinical validation. As a result, it remains challenging to evaluate how these simulations could be effectively implemented in routine practice and whether they can meaningfully inform decision-making in such settings.

### Regulatory, medico-legal, and ethical considerations

From a regulatory perspective, only a few digital twin applications are approved, including FFRCT and FEops LAA planning. Broader clinical use will require robust validation and evidence of impact on clinical outcomes.^51^ As digital twins evolve into clinical decision-support tools, medico-legal concerns arise. Incorrect predictions leading to adverse outcomes may raise liability issues.^52^ Clear communication of model limitations and adequate clinician training are essential to mitigate misuse. Ethical and data security concerns are also increasingly important, especially with cloud-based simulations and future integration of wearables or continuous monitoring.^53^ Ensuring patient consent, secure data handling, and compliance with privacy regulations (e.g. General Data Protection Regulation, Health Insurance Portability and Accountability Act) will be critical to maintaining public trust.

Although case-level costs for simulations such as FFRCT are reimbursed in several health systems and angiography-derived methods have minimal marginal cost, robust economic modelling for structural simulations (e.g. TAVR, LAAC) is lacking. Importantly, digital-twin outputs are decision-support tools, not deterministic solutions: proceduralists must interpret results in context, recognizing that parameters such as implant depth or commissural alignment may not be fully controllable during live procedures. Accordingly, human oversight, governance, and operator judgment remain indispensable, and ongoing research should address both cost-effectiveness and implementation frameworks to ensure responsible integration into practice.^5^ While the majority of current digital-twin applications are performed pre-procedurally, relying on CT or 3D echocardiography data, emerging approaches are exploring peri-procedural integration. These include fusion of preprocedural models with live fluoroscopy or echocardiography and the use of machine-learning surrogates for near-real-time haemodynamic prediction. Such developments may extend digital twins from planning tools into intra-procedural decision support, although these applications remain investigational.

### Future applications and development

Digital twin applications are poised to expand significantly across both coronary and structural interventions. In coronary disease, virtual PCI planning is undergoing clinical evaluation and may become a routine part of practice if ongoing studies confirm improvements in patient outcomes. In structural heart interventions—including TAVR, LAAC, and mitral therapies—simulation platforms are expected to proliferate beyond high-volume centres, supported by growing automation, improved usability, and increasing procedural standardization.

A central objective is the development of real-time digital twins that integrate intra-procedural data streams—such as fluoroscopic or echocardiographic imaging, intracardiac pressures, or pressure-volume loops—to dynamically inform procedural decisions. Achieving this vision will require substantial improvements in computational speed and responsiveness. Beyond procedural planning, future digital twins may evolve into longitudinal predictive tools, simulating disease progression, forecasting therapeutic response, and integrating physiologic inputs from wearables or remote monitoring systems.

Digital twins are also increasingly being incorporated into clinical trial workflows, where they enable simulation of device–tissue interactions, support virtual feasibility testing, and enhance procedural planning. Landmark studies such as PRECISE-TAVI and PREDICT-LAA have demonstrated that simulation-guided interventions can improve outcomes and reduce procedural variability. As their validation advances, digital twins may become integral to trial design, patient stratification, and regulatory approval pathways for emerging cardiovascular technologies.

### Artificial intelligence as an enabler of cardiac digital twins

AI is deeply embedded in the digital-twin pipeline, often operating invisibly to the end-user. AI algorithms drive automated segmentation of CT, angiography, and echocardiography, enabling rapid and reproducible anatomical modelling. For example, FFRCT relies on machine-learning methods to delineate coronary anatomy and predict pressure drops, while FEops HEARTguide integrates AI-assisted segmentation to construct finite-element models of the aortic root and left atrial appendage. Recent platforms such as LARALAB AI and Hi-D Imaging apply deep learning to accelerate chamber reconstruction and flow simulation. Beyond segmentation, AI supports surrogate modelling—machine-learning models trained on large CFD/FEA datasets that deliver near real-time haemodynamic predictions, substantially reducing computation time. Hybrid modelling approaches, which combine physics-based simulations with AI surrogates, are increasingly favoured to accelerate workflows and open the door to adaptive intra-procedural decision support. Finally, AI is being applied to predictive analytics, estimating patient-specific risks of conduction disturbances, paravalvular leak, or suboptimal post-PCI physiology. Together, these applications highlight AI as a critical enabler that transforms digital twins from technically feasible prototypes into clinically viable decision-support tools.

## Conclusion

Digital twin technology represents a paradigm shift in the approach to coronary and structural heart interventions within interventional cardiology. By creating personalized virtual replicas of patients, this technology offers unprecedented opportunities for enhanced precision in procedural planning and execution, improved prediction of outcomes, and the facilitation of individualized treatment strategies. The applications of digital twins are being actively explored and validated across the spectrum of these interventions. However, challenges related to data integration, model validation, computational resources, and ethical considerations remain important areas of focus. The future of digital twins in interventional cardiology is bright, with ongoing advancements in artificial intelligence, computational modelling, and real-time integration promising to further revolutionize the field and ultimately advance the goal of precision medicine for patients with coronary and structural heart disease.

## Data Availability

Data available upon request to the corresponding author.
